# Dynamic World, Near real-time global 10 m land use land cover mapping

**DOI:** 10.1038/s41597-022-01307-4

**Published:** 2022-06-09

**Authors:** Christopher F. Brown, Steven P. Brumby, Brookie Guzder-Williams, Tanya Birch, Samantha Brooks Hyde, Joseph Mazzariello, Wanda Czerwinski, Valerie J. Pasquarella, Robert Haertel, Simon Ilyushchenko, Kurt Schwehr, Mikaela Weisse, Fred Stolle, Craig Hanson, Oliver Guinan, Rebecca Moore, Alexander M. Tait

**Affiliations:** 1 Google, LLC, 1600 Amphitheatre Pkwy., Mountain View, CA 94043 USA; 2 National Geographic Society, 1145 17th St NW, Washington, DC 20036 USA; 3 World Resources Institute, 10 G St NE #800, Washington, DC 20002 USA; 4 Department of Earth & Environment, Boston University, 685 Commonwealth Avenue, Boston, MA 02215 USA

**Keywords:** Environmental impact, Ecological modelling

## Abstract

Unlike satellite images, which are typically acquired and processed in near-real-time, global land cover products have historically been produced on an annual basis, often with substantial lag times between image processing and dataset release. We developed a new automated approach for globally consistent, high resolution, near real-time (NRT) land use land cover (LULC) classification leveraging deep learning on 10 m Sentinel-2 imagery. We utilize a highly scalable cloud-based system to apply this approach and provide an open, continuous feed of LULC predictions in parallel with Sentinel-2 acquisitions. This first-of-its-kind NRT product, which we collectively refer to as Dynamic World, accommodates a variety of user needs ranging from extremely up-to-date LULC data to custom global composites representing user-specified date ranges. Furthermore, the continuous nature of the product’s outputs enables refinement, extension, and even redefinition of the LULC classification. In combination, these unique attributes enable unprecedented flexibility for a diverse community of users across a variety of disciplines.

## Abstract (summary)

**Table:** 

Measurement(s)	land use • land cover
Technology Type(s)	deep learning

## Background & Summary

Regularly updated global land use land cover (LULC) datasets provide the basis for understanding the status, trends, and pressures of human activity on carbon cycles, biodiversity, and other natural and anthropogenic processes^1–3^. Annual maps of global LULC have been developed by many groups. These maps include the National Aeronautics and Space Administration (NASA) MCD12Q1 500 m resolution dataset^4,5^ (2001–2018), the European Space Agency (ESA) Climate Change Initiative (CCI) 300 m dataset^6^ (1992–2018), and Copernicus Global Land Service (CGLS) Land Cover 100 m dataset^7,8^ (2015–2019). While widely used, many important LULC change processes are difficult or impossible to observe at a spatial resolution greater than 100 m and annual temporal resolution^9^, such as emerging settlements and small-scale agriculture (prevalent in the developing world) and early stages of deforestation and wetland/grassland conversion. Inability to resolve these processes introduces significant errors in our understanding of ecological dynamics and carbon budgets. Thus, there is a critical need for spatially explicit, moderate resolution (10–30 m/pixel) LULC products that are updated with greater temporal frequency.

Currently, almost all moderate resolution LULC products are available with only limited spatial and/or temporal coverage (e.g., USGS NLCD^10^ and LCMAP^11^) or via proprietary and/or closed products (e.g., BaseVue^12^, GlobeLand30^13^, GlobeLand10^14^) that are generally not available to support monitoring, forecasting, and decision making in the public sphere. A noteworthy exception is the recent iMap 1.0^15^ series of products available globally at a seasonal cadence with a 30 m resolution. Nonetheless, globally consistent, near real-time (NRT) mapping of LULC remains an ongoing challenge due to the tremendous computational and data storage requirements.

Simultaneous advances in large-scale cloud computing and machine learning algorithms in high-performance open source software frameworks (e.g., TensorFlow^16^) as well as increased access to satellite image collections through platforms such as Google Earth Engine^17^ have opened new opportunities to create global LULC datasets at higher spatial resolutions and greater temporal cadence than ever before. In this paper, we introduce a new NRT LULC dataset produced using a deep-learning modeling approach. Our model, which was trained using a combination of hand-annotated imagery and unsupervised methods, is used to operationally generate NRT predictions of LULC class probabilities for new and historic Sentinel-2 imagery using cloud computing on Earth Engine and Google Cloud AI Platform. These products, which we refer to collectively as Dynamic World, are available as a continuously updating Earth Engine Image Collection that enables users to leverage both class probabilities and multi-temporal results to track LULC dynamics in NRT and create custom products suited to their specific needs. We find that our model exhibits strong agreement with expert annotations for an unseen validation dataset, and though difficult to compare with existing products due to differences in temporal resolution and classification schemes, achieves better or comparable performance relative to other state-of-the-art global and regional products when compared to the same reference dataset.

## Methods

### Land Use Land Cover taxonomy

The classification schema or “taxonomy” for Dynamic World, shown in Table 1, was determined after a review of global LULC maps, including the USGS Anderson classification system^18^, ESA Land Use and Coverage Area frame Survey (LUCAS) land cover modalities^19^, MapBiomas classification^20^, and GlobeLand30 land cover types^13^. The Dynamic World taxonomy maintains a close semblance to the land use classes presented in the IPCC Good Practice Guidance (forest land, grassland, cropland, wetland, settlement, and other)^21^ to ensure easier application of the resulting data for estimating carbon stocks and greenhouse gas emissions. Unlike single-pixel labels, which are usually defined in terms of percent cover thresholds, the Dynamic World taxonomy was applied using “dense” polygon-based annotations such that LULC labels are applied to areas of relatively homogenous cover types with similar colors and textures.

**Table 1:** Dynamic World Land Use Land Cover (LULC) classification taxonomy.

Class ID	LULC Type	Description	Examples
0	Water	• Water is present in the image. • Contains little-to-no sparse vegetation, no rock outcrop, and no built-up features like docks. • Does not include land that can or has previously been covered by water.	• Rivers • Ponds & Lakes • Ocean • Flooded Salt Pans
1	Trees	• Any significant clustering of dense vegetation, typically with a closed or dense canopy. • Taller and darker than surrounding vegetation (if surrounded by other vegetation).	• Wooded vegetation • Dense green shrubs • Cluster of dense, tall vegetation within savannas • Plantations such as apples, bananas, citrus, and rubber • Swamp (dense/tall vegetation with no obvious water) • Any mix of the above • Any burned areas of the above
2	Grass	• Open areas covered in homogenous grasses with little to no taller vegetation. • Other homogenous areas of grass-like vegetation (blade-type leaves) that appear different from trees and shrubland. • Wild cereals and grasses with no obvious human plotting (i.e. not a structured field).	• Natural meadows and fields with sparse or no tree cover • Open savanna with little to no tree cover • Parks, golf courses, human manicured lawns, including large fields in urban settings like soccer and baseball. • Tree cut-throughs for power lines, gas etc. • Pastures • Reeds and marshes with no obvious flooding
3	Flooded vegetation	• Areas of any type of vegetation with obvious intermixing of water. • Do not assume an area is flooded if flooding is observed in another image. • Seasonally flooded areas that are a mix of grass/shrub/trees/bare ground.	• Flooded mangroves • Emergent vegetation
4	Crops	• Human planted/plotted cereals, grasses, and crops.	• Corn, wheat, soy, etc. • Hay and fallow plots of structured land
5	Shrub & Scrub	• Mix of small clusters of plants or individual plants dispersed on a landscape that shows exposed soil and rock. • Scrub-filled clearings within dense forests that are clearly not taller than trees. Appear grayer/browner due to less dense leaf cover.	• Moderate to sparse cover of bushes, shrubs, and tufts of grass • Savannas with very sparse grasses, trees, or other plants
6	Built area	• Clusters of human-made structures or individual very large human-made structures. • Contained industrial, commercial, and private building, and the associated parking lots. • A mixture of residential buildings, streets, lawns, trees, isolated residential structures or buildings surrounded by vegetative land covers. • Major road and rail networks outside of the predominant residential areas. • Large homogeneous impervious surfaces, including parking structures, large office buildings, and residential housing developments containing clusters of cul-de-sacs.	• Cluster of houses, can include smalls lawns or small patches of trees can be included • Dense villages, town, and cityscape (buildings and roads together) • Clusters of paved roads and large highways • Asphalt and other human-made surfaces
7	Bare ground	• Areas of rock or soil containing very sparse to no vegetation. • Large areas of sand and deserts with no to little vegetation. • Large individual or dense networks of dirt roads.	• Exposed rock • Exposed soil • Desert and sand dunes • Dry salt flats and salt pans • Dried lake bottoms • Mines • Large empty lots in urban areas
8	Snow & Ice	• Large homogenous areas of thick snow or ice, typically only in mountain areas or highest latitudes. • Large homogenous areas of snowfall.	• Glaciers • Permanent snowpack • Snowfall

Definitions and examples were provided as part of annotator reference materials, along with descriptions of colors and patterns typically associated with each LULC type.

### Training dataset collection

Our modeling approach relies on semi-supervised deep learning and requires spatially dense (i.e., ideally wall-to-wall) annotations. To collect a diverse set of training and evaluation data, we divided the world into three regions: the Western Hemisphere (160°W to 20°W), Eastern Hemisphere-1 (20°W to 100°E), and Eastern Hemisphere-2 (100°E to 160°W). We further divided each region by the 14 RESOLVE Ecoregions biomes^22^. We collected a stratified sample of sites for each biome per region based on NASA MCD12Q1 land cover for 2017^4^. Given the availability of higher-resolution LULC maps in the United States and Brazil, we used the NLCD 2016^10^ and MapBiomas 2017^20^ LULC products respectively in place of MODIS products for stratification in these two countries.

At each sample location, we performed an initial selection of Sentinel-2 images from 2019 scenes based on image cloudiness metadata reported in the Sentinel-2 tile’s QA60 band. We further filtered scenes to remove images with many masked pixels. We finally extracted individual tiles of 510 × 510 pixels centered on the sample sites from random dates in 2019. Tiles were sampled in the UTM projection of the source image and we selected one tile corresponding to a single Sentinel-2 ID number and single date.

Further steps were then taken to obtain an “as balanced as possible” training dataset with respect to the LULC classifications from the respective LULC products. In particular, for each Dynamic World LULC category contained within a tile, the tile was labeled to be high, medium, or low in that category. We then selected an approximately equal number of tiles with high, medium or low category labels for each category.

**Fig. 1:**
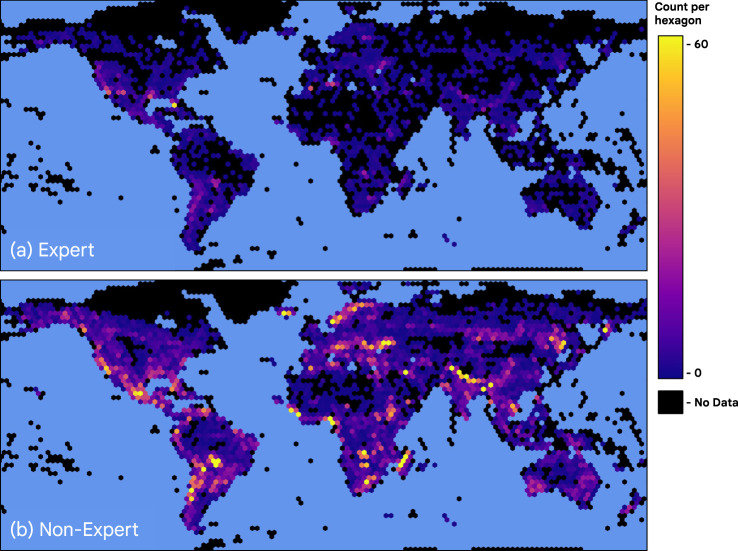
Global distribution of annotated Sentinel-2 image tiles used for model training and periodic testing (neither including 409 validation tiles). (**a**) 4,000 tiles interpreted by a group of 25 experts (**b**) 20,000 tiles interpreted by a group of 45 non-experts. Hexagons represent approximately 58,500 km^2^ areas and shading corresponds to the count of annotated tile centroids per hexagon.

To achieve a large dataset of labeled Sentinel-2 scenes, we worked with two groups of annotators. The first group included 25 annotators with previous photo-interpretation and/or remote sensing experience. The expert group labeled approximately 4,000 image tiles (Fig. 1a), which were then used to train and measure the performance and accuracy of a second “non-expert” group of 45 additional annotators who labeled a second set of approximately 20,000 image tiles (Fig. 1b). A final validation set of 409 image tiles were held back from the modeling effort and used for evaluation as described in the Technical Validation section. Each image tile in the validation set was annotated by three experts and one non-expert to facilitate cross-expert and expert/non-expert QA comparisons.

**Fig. 2:**
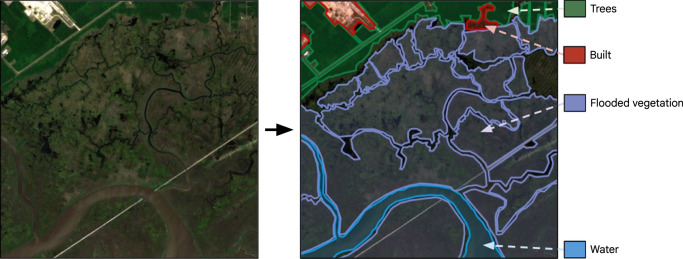
Sentinel-2 tile and example reference annotation provided as part of interpreter training. This example was used to illustrate the Flooded vegetation class, which is distinguished by small “mottled” areas of water mixed with vegetation near a riverbed. Also note that some areas of the tile are left unlabeled.

All Dynamic World annotators used the Labelbox platform^23^, which provides a vector drawing tool to mark the boundaries of feature classes directly over the Sentinel-2 tile (Fig. 2). We instructed both expert and non-expert annotators to use dense markup instead of single pixel labels with a minimum mapping unit of 50 × 50 m (5 × 5 pixels). For water, trees, crops, built area, bare ground, snow & ice, and cloud, this was a fairly straightforward procedure at the Sentinel-2 10 m resolution since these feature classes tend to appear in fairly homogenous agglomerations. Shrub & scrub and flooded vegetation classes proved to be more challenging as they tended not to appear as homogenous features (e.g. mix of vegetation types) and have variable appearance. Annotators used their best discretion in these situations based on the guidance provided in our training material (i.e. descriptions and examples in Table 1). In addition to the Sentinel-2 tile, annotators had access to a matching high-resolution satellite image via Google Maps and ground photography via Google Street View from the image center point. We also provided the date and center point coordinates for each annotation. All annotators were asked to label at least 70% of a tile within 20 to 60 minutes and were allowed to skip some tiles to best balance their labeling accuracy with their efficiency.

### Image preprocessing

**Fig. 3:**
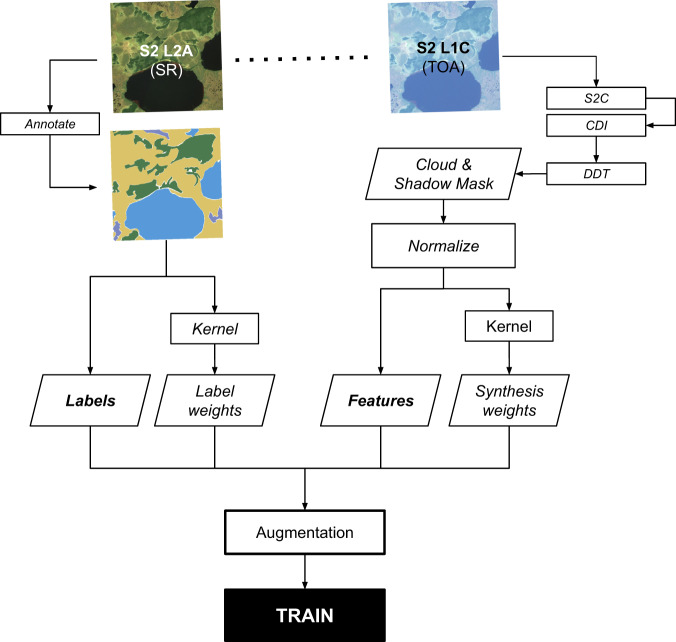
Training inputs workflow. Annotations created using Sentinel-2 Level 2 A Surface Reflectance imagery are paired with masked and normalized Sentinel-2 Level 1 C Top of Atmosphere imagery, and inputs are augmented to create training inputs used for modeling. Cloud and shadow masking involves a three-step process that combines the Sentinel-2 Cloud Probability (S2C) product with the Cloud Displacement Index (CDI), which is used to correct over-masking of bright non-cloud targets” and directional distance transform (DDT), which is used to remove the expected path of shadows based on sun-sensor geometry.

We prepared Sentinel-2 imagery in a number of ways to accommodate both annotation and training workflows. An overview of the preprocessing workflow is shown in Fig. 3.

For training data collection, we used the Sentinel-2 Level-2A (L2A) product, which provides radiometrically calibrated surface reflectance (SR) processed using the Sen2Cor software package^24^. This advanced level of processing was advantageous for annotation, as it attempts to remove inter-scene variability due to solar distance, zenith angle, and atmospheric conditions. However, systematically produced Sentinel-2 SR products are currently only available from 2017 onwards. Therefore, for our modeling approach, we used the Level-1C (L1C) product, which has been generated since the beginning of the Sentinel-2 program in 2015. The L1C product represents Top-of-Atmosphere (TOA) reflectance measurements and is not subject to a change in processing algorithm in the future. We note that for any L2A image, there is a corresponding L1C image, allowing us to directly map annotations performed using L2A imagery to the L1C imagery used in model training. All bands except for B1, B8A, B9, and B10 were kept, with all bands bilinearly upsampled to 10 m for both training and inference.

In addition to our preliminary cloud filtering in training image selection, we adopted and applied a novel masking solution that combines several existing products and techniques. Our procedure is to first take the 10 m Sentinel-2 Cloud Probability (S2C) product available in Earth Engine^25^ and join it to our working set of Sentinel-2 scenes such that each image is paired with the corresponding mask. We compute a cloud mask by thresholding S2C using a cloud probability of 65% to identify pixels that are likely obscured by cloud cover. We then apply the Cloud Displacement Index (CDI) algorithm^26^ and threshold the result to produce a second cloud mask, which is intersected with the S2C mask to reduce errors of commission by removing bright non-cloud targets based on Sentinel-2 parallax effects. We finally intersect this sub-cirrus mask with a threshold on the Sentinel-2 cirrus band (B10) using the thresholding constants proposed for the CDI algorithm^26^, and take a morphological opening of this as our cloudy pixel mask. This mask is computed at 20 m resolution.

In order to remove cloud shadows, we extend the cloudy pixel mask 5 km in the direction opposite the solar azimuthal angle using the scene level metadata “SOLAR_AZIMUTH_ANGLE” and a directional distance transform (DDT) operation in Earth Engine. The final cloud and shadow mask is resampled to 100 m to decrease both the data volume and processing time. The resulting mask is applied to Sentinel-2 images used for training and inference such that unmasked pixels represent observations that are likely to be cloud- and shadow-free.

The distribution of Sentinel-2 reflectance values are highly compressed towards the low end of the sensor range, with the remainder mostly occupied by high return phenomena like snow and ice, bare ground, and specular reflection. To combat this imbalance, we introduce a normalization scheme that better utilizes the useful range of Sentinel-2 reflectance values for each band. We first log-transform the raw reflectance values to equalize the long tail of highly reflective surfaces, then remap percentiles of the log-transformed values to points on a sigmoid function. The latter is done to bound on (0, 1) without truncation, and condenses the extreme end members of reflectances to a smaller range.

To account for an annotation skill differential between the non-expert and expert groups, we one-hot encode the labeled pixels, and smooth them according to the confidence in a binary label of the individual annotator (expert/non-expert): this is effectively linearly interpolating the distributions per-pixel from their one-hot encoding (i.e. a vector of binary variables for each class label) to uniform probability. We used 0.2 for experts, and 0.3 for non-experts (i.e. ~82% confidence on the true class for experts and ~73% confidence on the true class for the non-expert. We note that these values approximately mirror the Non-Expert to Expert Consensus agreement as discussed in the Technical Validation section). This is akin to standard label-smoothing^27,28^, with the addition that the degree of smoothing is associated with annotation confidence.

We generate a pair of weights for each pixel in an augmented example designed to compensate for class imbalance across the training set and weight high-frequency spatial features at the inputs during “synthesis” (discussed further in the following section). We also include a weight per pixel designed to attenuate labels in the center of labeled polygons where human annotators often missed small details using a simple edge finding kernel.

We finally perform a series of augmentations (random rotation and random per-band contrasting) to our input data to improve generalizability and performance of our model. These augmentations are applied four times to each example to yield our final training dataset of examples paired with class distributions, masks, and weights (Fig. 3).

### Model training

Our broad approach to transferring the supervised label data to a system that could be applied globally was to train a Fully Convolutional Neural Network (FCNN)^29^. Conceptually, this approach transforms pre-processed Sentinel-2 optical bands to a discrete probability distribution of the classes in our taxonomy on the basis of spatial context. This is done per-image with the assumption that sufficient spatial and spectral context is available to recover one of our taxonomic labels at a pixel. There are a few notable benefits to such an approach: namely that given the generalizability of modern deep neural networks, it is possible, as we will show, to produce a single model that achieves acceptable agreement with hand-digitized expert annotations globally. Furthermore, since model outputs are generated from a single image and a single model, it is straightforward to scale as each Sentinel-2 L1C image need only be observed once.

**Fig. 4:**
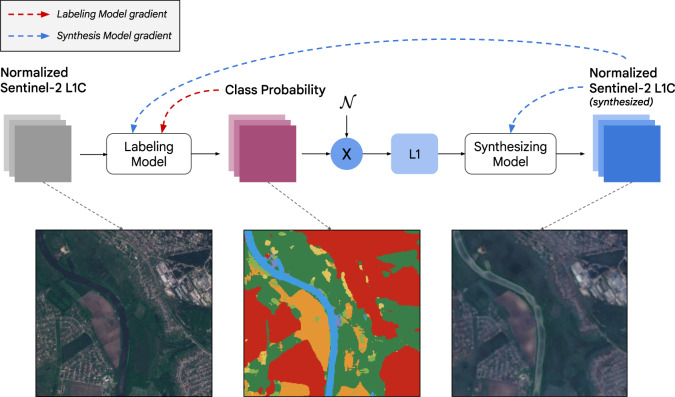
Training protocol used to recover the labeling model. The bottom row shows the progression from a normalized Sentinel-2 L1C image, to class probabilities, to synthesized Sentinel-2. The dashed red and blue arrows show how the labeling model is optimized with respect to both the class probability and synthesis pathway, and the synthesis model is optimized only with respect to the synthesized imagery. The example image is retrieved from Earth Engine using ee.Image(‘GOOGLE/DYNAMICWORLD/V1/20190517T083601_20190517T083604_T37UET’).

Although applying CNN modeling, including FCNN, to recover LULC is not a new idea^30–32^, we introduce a number of novel innovations that achieve state-of-the-art performance on LULC globally with a neural network architecture almost 100x smaller than architectures used for semantic segmentation or regression of ground-level camera imagery (specifically compared to U-Net^33^ and DeepLab v3+^34^ architectures). Our approach also leverages weak supervision by way of a synthesis pathway: this pathway includes a replica of the labeling model architecture that learns a mapping from estimated probabilities back to the input reflectances, in a way, a reverse LULC classifier that offers both multi-tasking and a constraint to overcome deficiencies in human labeling (Fig. 4).

### Near real-time inference

**Fig. 5:**
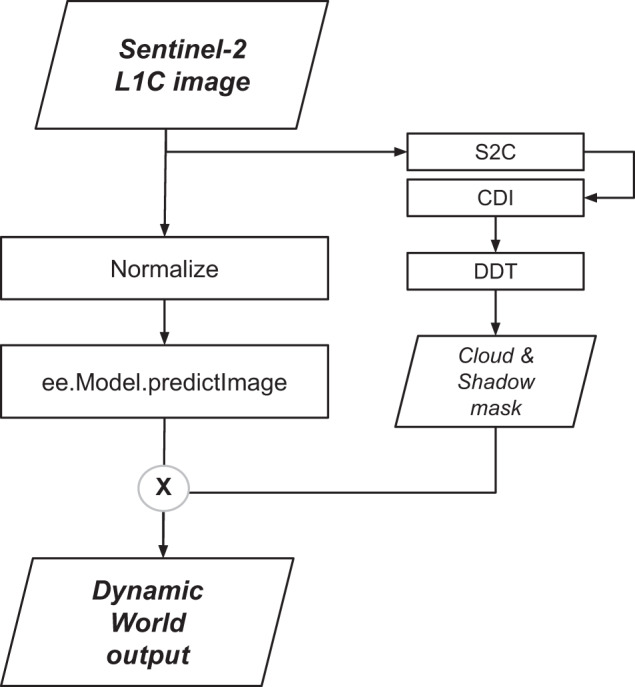
Near-Real-Time (NRT) prediction workflow. Input imagery is normalized following the same protocol used in training and the trained model is applied to generate land cover predictions. Predicted results are masked to remove cloud and cloud shadow artifacts using Sentinel-2 cloud probabilities (S2C), the Cloud Displacement Index (CDI) and a directional distance transform (DDT), then added to the Dynamic World image collection.

Using Earth Engine in combination with Cloud AI Platform, it is possible to handle enormous quantities of satellite data and apply custom image processing and classification methods using a simple scaling paradigm (Fig. 5). To generate our NRT products, we apply the normalization described earlier to the raw Sentinel-2 L1C imagery and pass all normalized bands except B1, B8A, B9 and B10 after bilinear upscaling to ee.Model.predictImage. This output is then masked using our cloud mask derived from the unnormalized L1C image. Creation of these images is triggered automatically when new Sentinel-2 L1C and S2C images are available. The NRT collection is continuously updated with new results. For a full Sentinel-2 tile (roughly 100 km x 100 km), predictions are completed on the order of 45 minutes. In total, we evaluate ~12,000 Sentinel-2 scenes per day, processing half on average due to a filter criteria on the CLOUDY_PIXEL_PERCENTAGE metadata of 35%. A new Dynamic World LULC image is processed approximately every 14.4 s.

## Data Records

The Dynamic World NRT product is available for the full Sentinel-2 L1C collection from 2015-06-27 to present. The revisit frequency of Sentinel-2 is between 2–5 days depending on latitude, though Dynamic World imagery is produced at about half this frequency (across all latitudes) given the aforementioned 35% filter on the CLOUDY_PIXEL_PERCENTAGE Sentinel-2 L1C metadata.

**Fig. 6:**
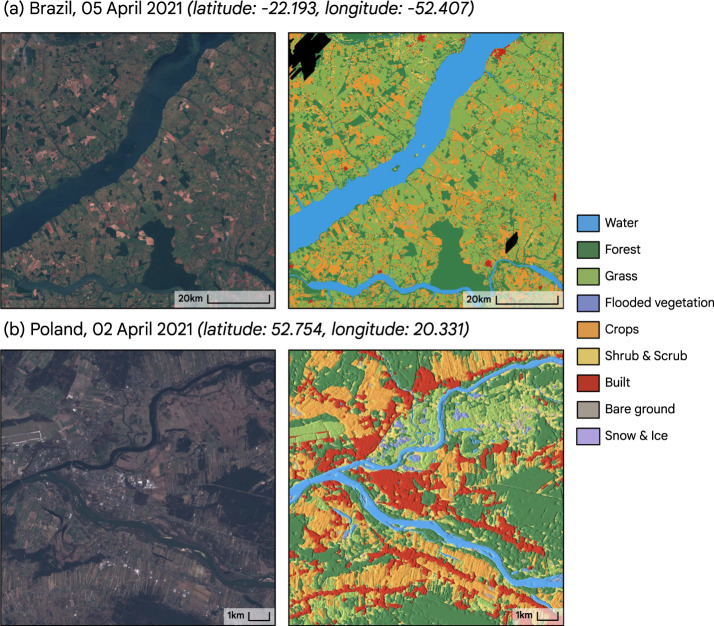
Examples of Sentinel-2 imagery (RGB) and corresponding Dynamic World NRT products for April 2021. Location coordinates reported for image centroid. (**a**) Brazil, ee.Image(‘GOOGLE/DYNAMICWORLD/V1/20210405T134209_20210405T134208_T22KCA’) and corresponding Dynamic World labels. (**b**) Poland, zoomed view of ee.Image(‘GOOGLE/DYNAMICWORLD/V1/20210402T095029_20210402T095027_T34UDD’) and corresponding Dynamic World product with a hillshade on the Top-1 confidence class applied to the categorical labels, revealing features not normally visible with discrete valued LULC maps.

The NRT product is hosted as an Earth Engine Image Collection under the collection ID “GOOGLE/DYNAMICWORLD/V1”. This is referenced in either the Earth Engine Python or JavaScript client library with ee.ImageCollection('GOOGLE/DYNAMICWORLD/V1') and in the Earth Engine data catalog at https://developers.google.com/earth-engine/datasets/catalog/GOOGLE_DYNAMICWORLD_V1^35^. The images in this collection have names matching the individual Sentinel-2 L1C asset IDs from which they were derived, e.g. a Sentinel-2 L1C image accessed in Earth Engine with ee.Image('COPERNICUS/S2/20160711T084022_20160711T084751_T35PKT') has a matching Dynamic World LULC product in ee.Image('GOOGLE/DYNAMICWORLD/V1/20160711T084022_20160711T084751_T35PKT') as in Fig. 6. Each image in the collection has bands corresponding to Table 2. Probability bands (all except the “label” band) sum to 1. Each image in the collection has additional metadata corresponding to Table 3.

**Table 2:** Bands of the images in the “GOOGLE/DYNAMICWORLD/V1” collection.

Index	Band Name	Description	Data Type	Range
0	water	Estimated probability of complete coverage by water.	double	(0, 1)
1	trees	Estimated probability of complete coverage by trees.	double	(0, 1)
2	grass	Estimated probability of complete coverage by grass.	double	(0, 1)
3	flooded_vegetation	Estimated probability of complete coverage by flooded vegetation.	double	(0, 1)
4	crops	Estimated probability of complete coverage by crops.	double	(0, 1)
5	shrub_and_scrub	Estimated probability of complete coverage by shrub and scrub.	double	(0, 1)
6	built	Estimated probability of complete coverage by built area.	double	(0, 1)
7	bare	Estimated probability of complete coverage by bare ground.	double	(0, 1)
8	snow_and_ice	Estimated probability of complete coverage by snow and ice.	double	(0, 1)
9	label	Index of the band with the highest estimated probability.	unsigned byte	[0, 8]

**Table 3:** Metadata of the images in the “GOOGLE/DYNAMICWORLD/V1” collection.

Name	Description	Data Type
system:index	The part of the path of the image in the collection following the final forward slash. This matches the system:index of the Sentinel-2 L1C image from which this image was derived.	string
system:time_start	The average acquisition time of pixels in this image in milliseconds since the Unix epoch. This matches the system:time_start of the Sentinel-2 L1C image from which this image was derived.	long integer
system:footprint	A geometry bounding the image data.	geometry
system:asset_size	The size in bytes of the image data as stored.	long integer
dynamicworld_algorithm_version	The version string uniquely identifying the Dynamic World model and inference process used to produce the image.	string
qa_algorithm_version	The version string uniquely identifying the cloud masking process used to produce the image.	string

Our 409-tile test dataset, including expert consensus annotations and corresponding Dynamic World estimated probabilities and class labels for each 5100 m × 5100 m tile are archived in Zenodo at the following 10.5281/zenodo.4766508^36^. The training dataset has been archived in PANGAEA in a separate repository: 10.1594/PANGAEA.933475^37^. The training and test data collected for Dynamic World are also available as Earth Engine Image Collection and can be accessed with:


ee.ImageCollection(‘projects/wri-datalab/dynamic_world/v1/DW_LABELS’).


## Technical Validation

We used several different approaches to characterize the quality of our NRT products. We first compared expert and non-expert annotations to establish baseline agreement across human interpreters. This is particularly relevant in understanding the quality of 20,000 training tiles that were annotated by non-experts. We then compared expert reference annotations with Dynamic World products and to existing national and global products produced at an annual time step. We note that, for all comparisons with Dynamic World products, we ran the trained Dynamic World model directly on the Sentinel-2 imagery in the test tile and applied our cloud mask in order to benchmark the NRT results for the reference image date.

**Fig. 7:**
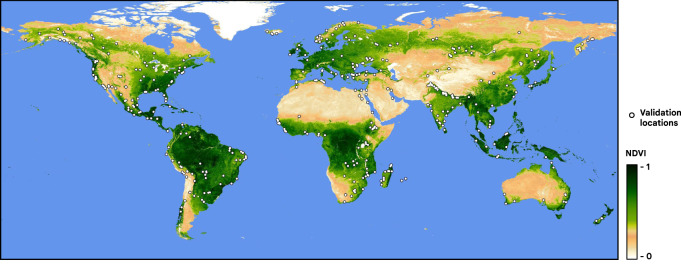
409 annotated Sentinel-2 tile centers in the test dataset, shown as white points overlaid on a 2019 MODIS NDVI composite to show global distribution of vegetated areas.

To create a balanced validation set, we randomly extracted ten image-markup pairs per biome per hemisphere from the existing markups: 140 from the 14 biomes in the Western Hemisphere, 130 from the 13 biomes in Eastern Hemisphere-1, and another 140 from the 14 biomes in Eastern Hemisphere-2. Each tile was independently labeled by three annotators from the expert group and by a member of the non-expert group such that we had four different sets of annotations for each validation tile. In total, this process produced 1636 tile annotations over 409 Sentinel-2 tiles (Fig. 7), and these tiles were excluded from training and online validation.

Because new Dynamic World classifications are generated for each individual Sentinel-2 image and the quality of these classifications is expected to vary spatially and temporally as a function of image quality, it is difficult to provide design-based measures of accuracy that are representative of the full (and continuously updating) collection. Therefore, we focus instead on using the multiple annotations for each validation tile as a means to characterize both the quality and agreement of annotations themselves, as well as the ability of our NRT model to generalize to new (unseen) images at inference time.

Annotations were combined in three different ways to measure (1) agreement between expert and non-expert labels, (2) expert-to-expert consistency, and (3) agreement between machine labeling and multi-expert consensus under several expert voting schemes.The four voting schemes considered were *Three Expert Strict agreement*, where all three experts had an opinion and all three agreed on feature class; *Expert Consensus*, where all three experts agreed, or where two experts agreed and the third had no opinion, or where one expert had an opinion and the other two did not; *Expert Majority*, where at least two experts agreed on feature class, or where one expert had an opinion and the other two did not; *Expert Simple Majority*, where at least two experts agreed and at least two agreed on feature class.

### Comparison of expert and non-expert annotations

To assess the quality of non-expert annotations, which comprise the majority of our training dataset, we directly compared rasterized versions of hand-digitized expert and non-expert annotations for our validation sample. Though these validation images were not used as part of model training, this comparison highlights strengths and potential weaknesses of the training set. We summarize the agreement between non-experts and experts for different voting schemes in Table 4 and show the full confusion matrix of Non-Experts to Expert Consensus in Table 5.

**Table 4:** Agreement between non-experts and expert voting schemes.

	Three Expert Strict	Expert Consensus	Expert Majority	Simple Expert Majority
Non-Expert Agreement	91.5%	77.8%	75.2%	81.4%

**Table 5:** Per-pixel confusion matrix of Non-Experts to Expert Consensus.

	Expert Consensus											
		Water	Trees	Grass	Flooded Vegetation	Crops	Shrub & Scrub	Built Area	Bare Ground	Snow & Ice	Cloud	Precision/User’s
Non-Experts	Water	7814103	36668	21205	58174	193344	18921	17533	68692	3688	3002	94.90%
	Trees	237858	16664442	499046	301831	657282	1834363	133325	71652	18987	19774	81.50%
	Grass	9921	60697	540597	55648	265055	113899	14984	18240	550	1407	50.00%
	Flooded Vegetation	255910	107576	23721	879482	40311	33552	9357	23270	0	0	64.00%
	Crops	13392	150401	258153	7920	12567377	223368	92983	88258	0	5141	93.70%
	Shrub & Scrub	72746	1748087	1008622	519267	1656386	5143322	245498	1996929	137415	12010	41.00%
	Built Area	10716	222831	40154	2028	324127	59196	6224787	35812	866	1510	89.90%
	Bare Ground	22661	25522	23480	4482	268845	257830	22460	1036373	49328	4090	60.40%
	Snow & Ice	2644	59334	539	687	73	21734	0	15917	1388854	11	93.20%
	Cloud	3033	8988	198	264	19887	244	3740	393	3955	161082	79.80%
	**Recall/Producer’s**	**92.60%**	**87.30%**	**22.40%**	**48.10%**	**78.60%**	**66.70%**	**92.00%**	**30.90%**	**86.60%**	**80.20%**	**77.80%**

Note that cloud is included as both sets of annotations include this label (n = 58,963,662).

Agreement for all comparisons was greater than 75%, suggesting fairly consistent labeling across different levels of expertise. As would be expected, the *Three Expert Strict* set shows the highest overlap with the *Non-Expert* set (91.5%), as only the pixel labels with the highest confidence amongst expert annotators remain.

### Comparison of Dynamic World predictions with expert annotations

To assess the model’s ability to generalize to new images, the trained Dynamic World model was applied to the 409 test tiles and the class with the highest probability (or “Top-1” label) was compared to the four expert voting schemes. Neither the validation images, nor other images from the same locations were available to the model during training. Thus, this assessment quantifies how well the model performs when applied outside the training domain. The results of these comparisons are shown in Tables 6–9.

**Table 6:** Confusion matrix of Dynamic World to Three Expert Strict, i.e. valid where all three experts labeled and all agreed (n = 27,841,623).

	Three Expert Strict										
		Water	Trees	Grass	Flooded Vegetation	Crops	Shrub & Scrub	Built Area	Bare Ground	Snow/Ice	Precision/User’s:
Dynamic World	Water	5964550	1450	0	17674	51640	125	5212	7713	2	98.60%
	Trees	18510	7966289	118152	97159	656797	238465	8677	668	0	87.50%
	Grass	24	4641	223688	8704	478891	38111	1046	20504	0	28.80%
	Flooded Veg.	1680	1600	2185	275367	36005	3283	32	79	0	86.00%
	Crops	262	11326	8093	426	5316019	126244	7389	5527	0	97.10%
	Shrub & Scrub	408	121316	16676	497	309762	913085	14460	30428	0	64.90%
	Built Area	63	4416	521	0	73663	3385	2506552	2171	0	96.70%
	Bare Ground	126219	392	103	0	171023	155783	77606	901851	0	62.90%
	Snow & Ice	49541	55081	0	0	25229	466	10457	8959	537301	78.20%
	Recal/Producer’sl:	96.80%	97.50%	60.60%	68.90%	74.70%	61.70%	95.30%	92.20%	100.00%	88.4%

**Table 7:** Confusion matrix of Dynamic World to Expert Consensus, i.e. valid where at least two experts labeled and all agreed in any case (n = 68,137,571).

	Expert Consensus										
		Water	Trees	Grass	Flooded Vegetation	Crops	Shrub & Scrub	Built Area	Bare Ground	Snow/Ice	Precision/User’s:
Dynamic World	Water	7664249	47476	34405	160034	333689	54613	45573	112658	4178	90.60%
	Trees	121205	17522174	1019096	803380	2565217	2529992	281318	120507	8921	70.20%
	Grass	5956	83205	876142	149792	1343601	311448	39657	101129	695	30.10%
	Flooded Veg.	51371	68818	45450	722106	120045	56370	6860	35856	6	65.20%
	Crops	21083	93924	139766	35422	9841373	574660	126895	241771	38	88.90%
	Shrub & Scrub	17666	628594	380724	75929	1220212	3552589	151919	440744	29373	54.70%
	Built Area	10375	146794	55121	3930	610401	94431	6489015	75899	744	86.70%
	Bare Ground	171029	15374	28976	8811	313838	661030	183342	2214615	42538	60.80%
	Snow & Ice	68277	195648	8649	550	59474	104295	14122	122907	1417512	71.20%
	Recall/Producer’s:	94.30%	93.20%	33.80%	36.80%	60.00%	44.70%	88.40%	63.90%	94.20%	73.80%

**Table 8:** Confusion matrix of Dynamic World to Expert Majority, i.e. valid where, amongst labels, there was consensus or only one expert labeled (n = 78,916,422).

	Expert Majority										
		Water	Trees	Grass	Flooded Vegetation	Crops	Shrub & Scrub	Built Area	Bare Ground	Snow/Ice	Precision/User’s:
Dynamic World	Water	7801513	49529	39511	187484	511166	59061	46461	197843	4178	87.70%
	Trees	127510	20225220	1280463	963384	2835774	3215029	293635	150630	8956	69.50%
	Grass	6465	135888	1415436	170294	1917310	405560	41812	151648	695	33.30%
	Flooded Veg.	54365	73337	56852	764482	133162	72474	6878	40949	6	63.60%
	Crops	23790	144438	261750	36363	10821384	707154	134486	328916	38	86.90%
	Shrub & Scrub	18649	1034643	551074	99947	1476117	4498630	157166	708463	32079	52.50%
	Built Area	11187	156117	57647	3935	666712	104009	6620303	82974	744	85.90%
	Bare Ground	178304	16203	40275	8835	400529	1022049	198274	2722023	48658	58.70%
	Snow & Ice	68319	199018	8656	550	59786	109192	14527	214993	1422556	67.80%
	Recall/Producer’s:	94.10%	91.80%	38.10%	34.20%	57.50%	44.10%	88.10%	59.20%	93.70%	71.30%

**Table 9:** Confusion matrix of Dynamic World to Expert Simple Majority, i.e. valid where at least one expert labeled and all agreed in any case (n = 57,707,212).

	Expert Simple Majority										
		Water	Trees	Grass	Flooded Vegetation	Crops	Shrub & Scrub	Built Area	Bare Ground	Snow/Ice	Precision/User’s:
Dynamic World	Water	7203733	12969	9542	82175	422487	16660	15451	116578	57	91.40%
	Trees	51183	15906784	596718	512895	1728587	1589558	80497	50509	492	77.50%
	Grass	1066	77515	1030113	72589	1451071	213904	13709	91378	0	34.90%
	Flooded Veg.	12885	15142	18924	521553	91294	33735	600	10042	0	74.10%
	Crops	3830	78317	153125	12494	9204745	469898	37157	127148	0	91.30%
	Shrub & Scrub	3231	663092	248003	36709	958322	2969428	64467	389238	5807	55.60%
	Built Area	2150	36312	6089	68	325235	24489	4931216	22913	0	92.20%
	Bare Ground	142656	2294	15237	203	338099	732591	132422	2124769	9991	60.70%
	Snow & Ice	53479	128014	9	0	49884	26204	12566	134024	978892	70.80%
	Recall/Producer’s:	96.40%	94.00%	49.60%	42.10%	63.20%	48.90%	93.30%	69.30%	98.40%	77.8%

We considered the Expert Consensus scheme to best balance “easy” labels (where many experts would agree) and “hard” labels (where labels would be arguably more ambiguous) and used this as our primary performance metric. Overall agreement between these single-image Dynamic World model outputs and the expert labels was observed to be 73.8%. Comparing this 73.8% to the non-expert to expert agreement of 77.8% in Table 5, we note the similarity of the predictions to the level of agreement amongst the labels themselves. Unsurprisingly the model achieved the highest agreement for classes where annotators were confident (water, trees, built area, snow & ice) but had greater difficulty for classes where the annotators were less confident (grass, flooded vegetation, shrub & scrub, and bare ground).

### Comparison of Dynamic World and other LULC datasets

**Fig. 8:**
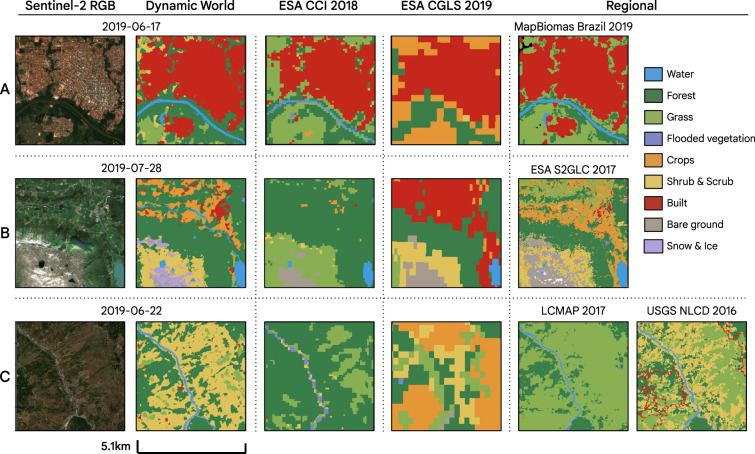
Visual comparison of Dynamic World (DW) to other global and regional LULC datasets for validation tile locations in (**A**) Brazil (−11.437°, −61.460°), (**B**) Norway, (61.724°, 6.484°), and (**C**) the United States (39.973°, −123.441°). Datasets used for comparison include 300 m European Space Agency (ESA) Climate Change Initiative (CCI); 100 m Copernicus Global Land Service (CGLS) ProbaV Land Cover dataset; 10 m ESA Sentinel-2 Global Land Cover (S2GLC) Europe 2019; 30 m MapBiomas Brazil dataset; and 30 m USGS National Land Cover Dataset (NLCD). Each map chip represents a 5.1 km by 5.1 km area with corresponding true-color (RGB) Sentinel-2 image shown in the first column. All products have been standardized to the same legend used for DW. Note differences in resolution as well as differences in the spatial distribution and coverage of land use land cover classes.

As a third point of comparison, we contextualize our results in terms of existing products. We qualitatively and quantitatively compared Dynamic World with other publicly available global and regional LULC datasets (Table 10). For each Dynamic World validation tile, we reprojected the compared dataset to the UTM zone of the tile, upsampled the data to 10 m using nearest-neighbor resampling, and extracted a tile matching the extent of the labeled validation tile. For regional LULC datasets, such as LCMAP, NLCD, and MapBiomas, we were limited to tiles located within the regional boundary (e.g., only 42 validation tiles are within the spatial coverage of MapBiomas). We note that in every case, some cross-walking was necessary to match the taxonomies to the Dynamic World LULC classification scheme. We show a visual comparison of Dynamic World to other products in Fig. 8.

**Table 10:** Comparison of Dynamic World to other LULC datasets in terms of temporal frequency, global coverage, agreement with our Expert Consensus test dataset, scale and Sentinel-2 tiles mapped.

Dataset	NRT	Global	Agreement	Scale (m)	Tiles
Mapbiomas Amazonia 2018 (N.Brazil, Venezuela, Peru, Bolivia)	No	No	54.8%	30	11
ESA S2GLC Europe 2019	No	No	59.2%	**10**	45
ESA CCI 2018	No	**Yes**	61.6%	300	409
ESA CGLS ProbaV 2019	No	**Yes**	66.3%	100	409
NLCD 2016 (30 m, CONUS + Alaska)	No	No	66.7%	30	56
Mapbiomas Brazil 2019	No	No	67.4%	30	20
LCMAP 2017 (30 m, CONUS only)	No	No	**75.0%**	30	48
Dynamic World (NRT)	**Yes**	**Yes**	73.8%	**10**	409

Bold values indicate top qualitative performance in each comparison category.

Measured against the expert consensus of annotations for the 409 global tiles, Dynamic World exceeded the agreement of all other LULC datasets except for the regional product LCMAP 2017 (Table 10). For the best global LULC product in our comparison study (ESA CGLS ProbaV 2019), Dynamic World achieved agreement at a higher spatial resolution (10 m vs 100 m) and improved agreement by 7.5%. For the current best regional product (LCMAP 2017), Dynamic World agreed 1.2% less with our expert consensus. We note that to perform the LCMAP comparison, we had to reduce our number of classes by combining grass and shrub & scrub as LCMAP does not separate these classes. When combining the Dynamic World grass and shrub & scrub classes, the agreement rises slightly to 74.2%, though LCMAP agreement was only validated against 11.7% of the tiles in a regional sample, and is an annual product not NRT. Further, direct comparison to ESA datasets are difficult due to the resolution differences, with 300 m more spatially generalized than 10 m. It is also important to note that the Dynamic World comparison to the annotated validation tile is for the same image date, while there may be a mismatch in dates when comparing to other LULC datasets. Thus, by characterizing the relative agreement of different datasets with hand-annotated labels for a specific Sentinel-2 image, these comparisons provide important insights into the value of NRT classification for capturing fine-grained spatial and temporal variability in LULC.

## Usage Notes

Extensions of the Dynamic World NRT collection offer new opportunities to create global analysis products at a speed, cost, and performance that is appropriate for a broad range of stakeholders, e.g. national or regional governments, civil society, and national and international research and policy organizations. It is our hope that Dynamic World and spatially consistent products like it can begin to make LULC and derived analysis globally equitable.

### Time series of class probabilities

Though we used Top-1 labels for validation and cross-dataset comparisons, Dynamic World includes class probabilities in addition to a single “best” label for each pixel (Table 2). While inclusion of class probabilities and other continuous metrics that characterize uncertainties in LULC classifications are becoming increasingly common (i.e. LCMAP cover confidence attributes^11^), Dynamic World is distinct in providing dense time series of class probabilities updated with a similar cadence to the acquisition of the source imagery itself.

**Fig. 9:**
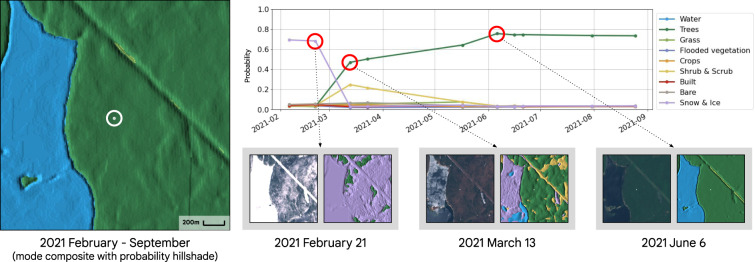
Example of Dynamic World mode composite (February - September 2021), time series of class probabilities for single pixel (location indicated by circled white point), and select Dynamic World predictions with corresponding single-date Sentinel-2 images for temperate deciduous forest in Massachusetts, USA (centered on latitude: 42.491°, longitude: −72.275°).

Rather than provide LULC labels that are intended to represent a multi-date time period, Dynamic World provides single-date snapshots that reflect the highly transitional and temporally dynamic nature of cover type probabilities. For example, in temperate regions that experience seasonal snow cover, a mode composite of Dynamic World labels reflects dominant tree and water cover types from February through September (Fig. 9). However, a time series of class probabilities for a pixel in an area of deciduous forest that is classified as “Trees” in the mode composite and during leaf-on conditions (e.g. June 6) is also classified as Snow & Ice when the ground is snow-covered (February 21) and has an increased Shrub & Scrub probability during early spring before leaf-out (March 13). This example illustrates the advantages of an instantaneous and probabilistic NRT classification approach, while also highlighting the challenges of standardizing validation metrics for a dynamic LULC dataset.

### Uncertainties

We find single-date Dynamic World classifications agree with the annotators nearly as well as the annotators agree amongst each other. The Dynamic World NRT product also achieves performance near, or exceeding many popular regional and global annual LULC products when compared to annotations for the same validation tiles. However, we have observed that performance varies spatially and temporally as a function of both the quality of S2 cloud masking and variability in land cover and condition.

**Fig. 10:**
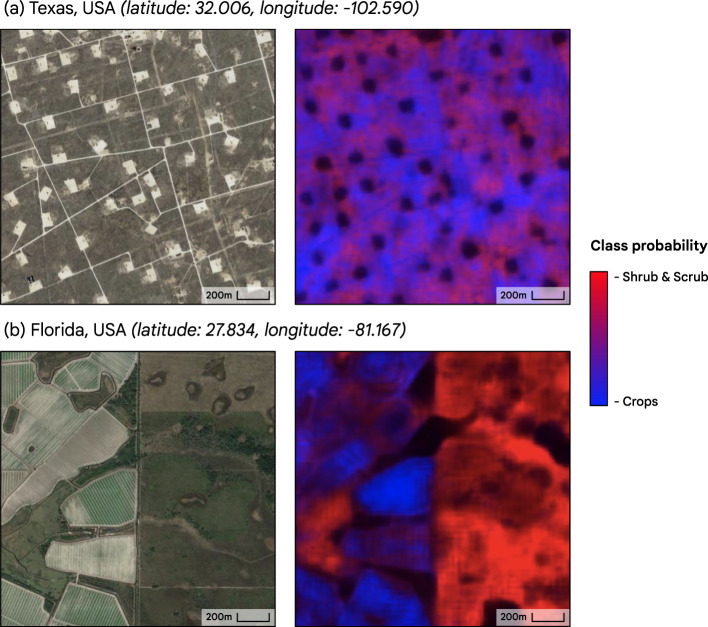
Demonstration of relative weakness exhibited in Dynamic World in separating arid shrubland from crops. (**a**) An oil field in Texas, USA; (**b**) Agricultural mosaic in Florida, USA. High resolution image shown for reference. Estimated class prediction probabilities scaled from [0, 1] with red corresponding to the maximum probability of the crops class and blue corresponding to the maximum probability of the shrub & scrub class. In arid shrubland, the estimated probabilities for shrub and crops are more similar (purple) than in temperate or other biomes. The probabilities were averaged per-pixel over July 2021 and the reference imagery was taken from the Google Maps Satellite layer.

Dynamic World tends to perform most strongly in temperate and tree-dominated biomes. Arid shrublands and rangelands were observed to present the greatest source of confusion specifically between crops and shrub. In Fig. 10, we demonstrate this phenomenon by observing that the maximum of estimated probabilities between crops and shrubs tends towards 0.5 in a sample of arid shrubland in Texas (seen by the low contrast purple coloring) even though this region does not contain cultivated land. By visual qualitative inspection, Dynamic World identifies grasslands better than the generally low agreement suggested by our Expert Consensus (30.1% for Dynamic World to 50% by non-experts, a 19.9% delta), and identifies crops more poorly than the generally high agreement suggested by our Expert consensus (88.9% by Dynamic World to 93.7% by non-experts, a 4.8% delta).

We also note that single-date classifications are highly dependent on accurate cloud and cloud shadow masking. Though we have implemented a fairly conservative masking process that includes several existing products and algorithms, missed clouds are typically misclassified as Snow & Ice and missed shadows as Water. However, because Dynamic World predictions are directly linked to individual Sentinel-2 acquisitions, these misclassifications can be identified by inspecting source imagery and resolved through additional filtering or other post-processing.

### Creating new products from the Dynamic World collection

**Fig. 11:**
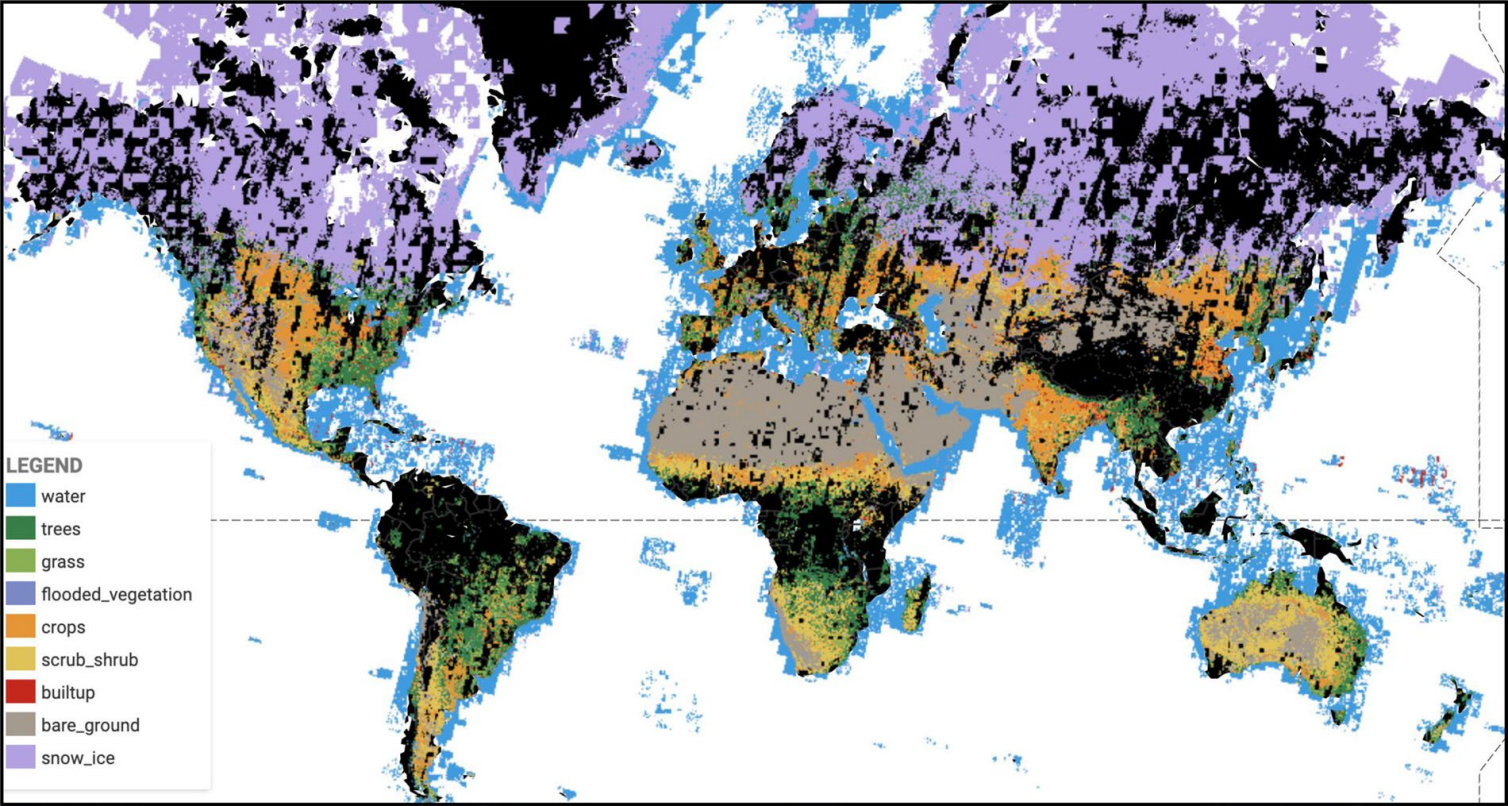
Mode composite of all Dynamic World NRT products from 2021-04-01 to 2021-05-01. Areas of black correspond to no data over land (due to cloud cover) with white corresponding to no data over water.

As a fundamentally NRT and continuous product, Dynamic World allows users to constrain observed data ranges and leverage the continuous nature of the outputs to characterize land conditions as needed for their specific interests and tasks. For example, we do not expect the prescriptiveness of the “label” band to be appropriate for all user needs. By applying a desired threshold or more advanced decision framework to the estimated probabilities, it is possible to customize a discrete classification as is appropriate for a user’s unique definitions or downstream task. Furthermore, users can aggregate NRT results to represent longer time periods. For example, one could create a monthly product as seen in Fig. 11 by mode-compositing the highest probability label over a one month period using a simple filterDate and mode in Earth Engine. It is also straightforward to generate a more traditional annual product by aggregating the estimated distributions for a given year or between the spring and autumn equinoxes to represent growing season cover only. Thus, unlike conventional map products, Dynamic World enables a greater degree of flexibility for users to generate custom aggregations and derivative products uniquely tailored to their needs and study areas.

### Quantifying accuracy of derived products

Rigorous assessment of map accuracy and good practices in estimating areas of mapped classes require probability sampling design that supports design-based inference of population-level parameters such as overall accuracy^38^. However, one of the fundamental requirements of design-based inference is a real, explicitly defined population, and in the case of map accuracy assessment, this population typically refers to a population of pixels included in a map and assigned different class labels^39^. Given that Dynamic World is a continuously updating image collection that can be post-processed into any number of different map products, the construction of a design-based sample would be dependent on the specific temporal aggregations and/or reclassifications performed by end-users.

In the assessments performed as part of our Technical Validation, we focus on agreement between reference annotations and our Top-1 NRT labels as our primary validation metric. While these agreement assessments support the general quality and utility of the Dynamic World dataset from the perspective of benchmarking, we note that our confusion matrices are not population confusion matrices and thus cannot be used to estimate population parameters. These matrices also do not account for model-based estimates of uncertainty, specifically class probability bands that characterize uncertainty in model predictions. While more rigorous characterization of model uncertainty could be achieved using model-based inference techniques^38^, we argue that this is less appropriate for products like Dynamic World that are intended to be further refined into more traditional map products that can be assessed using design-based methods.

As an example, a Dynamic World derived product was generated by simply averaging class probabilities and a proof-of-concept assessment was performed by the University of Maryland Global Land Analysis and Discovery Laboratory (UMD-GLAD) using a stratified random sampling strategy with a total of 19 strata based on a prototype 30 m UMD-GLAD LULC map. Fifty sampling units were randomly selected from each of the 19 strata. Reference data for interpretation and class assignment consisted of high resolution data from the Google Maps Satellite layer viewed in Google Earth and MODIS time-series NDVI. Each interpreted sampling unit was re-labeled with one of the eight DynamicWorld classes and all results were compared to the temporally aggregated DynamicWorld product. Results generally indicated higher accuracies in terms of precision/user’s accuracy and recall/producer’s accuracy for relatively stable LULC classes such as water and trees. However, mixed classes such as built area and shrub & scrub and classes such as bare ground, crop, grass, and flooded vegetation that represent transient states or exhibit greater temporal dynamics tended to show much lower accuracies. Some of these lower levels of agreement also reflect potential mismatches in class definitions that arise from the NRT nature of the Dynamic World classes, i.e. “Flooded vegetation” may characterize an ephemeral state that is different from a more traditional “wetland” categorization.

While this example provides one possible derived product and assessment useful for demonstration purposes, we intentionally do not provide a standard derivative map product of the Dynamic World dataset and instead encourage users, as is standard practice, to develop assessments of their unique derivative map products using tools such as Collect Earth^40^ designed for reference data collection and community standard guidance^41–43^. Reference sample design should reflect user-specified temporal aggregation (i.e., monthly, annual, multi-year) as well as any post-classification modifications to the original Dynamic World legend. There may also be interesting opportunities to compare Dynamic World NRT and derived products with existing reference samples (e.g., LCMAP), in which case accuracy results and area estimates should be computed using estimators that account for differences between the map used for sample stratification and the Dynamic World product being assessed.

## Data Availability

The Dynamic World NRT dataset has been made available as an Earth Engine Image Collection under “GOOGLE/DYNAMICWORLD/V1”. This is referenced in either the Earth Engine Python or JavaScript client library with: ee.ImageCollection(‘GOOGLE/DYNAMICWORLD/V1’). We provide a public web interface for rapid exploration of the dataset at: https://sites.google.com/view/dynamic-world/home. We also provide an example of accessing Dynamic World using the Earth Engine Code Editor in the following code snippet: https://code.earthengine.google.com/710e2ae9d03cd994c6e8dc9213257cbc. The Dynamic World model has been run for historic Sentinel-2 imagery and is being run for newly acquired Sentinel-2 imagery; users are therefore encouraged to work with outputs available in the NRT Image Collection available on Earth Engine. Nonetheless, to ensure reproducibility, we have archived the trained model, example code for running inference, and additional information on the model architecture in Zenodo at 10.5281/zenodo.5602141^44^.
